# Fibroblast-Like-Synoviocytes Mediate Secretion of Pro-Inflammatory Cytokines via ERK and JNK MAPKs in Ti-Particle-Induced Osteolysis

**DOI:** 10.3390/ma13163628

**Published:** 2020-08-17

**Authors:** Ashish Ranjan Sharma, Supriya Jagga, Chiranjib Chakraborty, Sang-Soo Lee

**Affiliations:** Institute For Skeletal Aging & Orthopedic Surgery, Hallym University-Chuncheon Sacred Heart Hospital, Chuncheon, Gangwon-do 24252, Korea; researchskeletal@gmail.com (A.R.S.); sjagga@bwh.harvard.edu (S.J.); drchiranjib@yahoo.com (C.C.)

**Keywords:** wear debris, fibroblast-like synoviocytes (FLS), MAPKs, pro-inflammatory cytokines

## Abstract

Biomaterials are designed to replace and augment living tissues in order to provide functional support to skeletal deformities. However, wear debris produced from the interfaces of metal implants initiates inflammatory bone loss, causing periprosthetic osteolysis. Lately, fibroblast-like synoviocytes (FLS) have been shown to play a role in wear-debris-induced osteolysis. Thus, here we have tried to understand the underlying mechanism of FLS involvement in wear-debris-induced osteolysis. Our results demonstrate that the effects of Ti particle (1:100 cell-to-Ti particle ratio) on FLS can induce Cox-2 expression and activate NFkB signaling. Moreover, the mRNA expression of pro-inflammatory cytokines such as IL-6, IL-8, IL-11, IL-1β, and TNFα was found to be elevated. However, among these pro-inflammatory cytokines, the mRNA and protein levels of only IL-6, IL-1β, and TNFα were found to be significantly higher. Ti particles activated extracellular signal-regulated kinase (ERK) and c-Jun N-terminal kinase (JNK) mitogen-activated protein kinases (MAPKs) as an early response in FLS. Co-inhibition of ERK and JNK signaling pathways by their specific inhibitors (PD9805 and SP600125, respectively) resulted in the suppression of mRNA and protein levels of IL-6, IL-1β, and TNFα in FLS. Taken together, targeting ERK and JNK MAPKs in FLS might provide a therapeutic option for reducing the secretion of bone-resorbing pro-inflammatory cytokines, thus preventing periprosthetic osteolysis.

## 1. Introduction

Degenerative skeletal diseases such as osteoarthritis, rheumatoid arthritis, and osteoporosis lead to skeletal disabilities and often require total joint arthroplasty (TJA) to provide restoration of function and pain relief. TJA is an overwhelming successful surgical intervention of modern medicine that is a remarkably effective and safe method of treatment [1,2]. Annually, millions of people (>1.3 million) undergo TJA [3]. However, within 10 years of surgery, up to 20% of these cases require a revision [4]. Moreover, as young and more active populations are undergoing TJA with insufficient implant durability, more TJAs revisions are expected [5]. Wearing of prosthetic implants with time is the major concern related to TJAs [6]. Particulate debris can be produced by diverse kinds of processes, including corrosion, micromotion, and oxidative reactions of implants [7]. The buildup of particulate debris from the interface of orthopedic implants can trigger biological response leading to aseptic loosening and immense bone loss, requiring revision of surgery for the patients [8]. The size of osteolytic lesions and the subsequent risks are extensively reliant on the arrangement of the implants and the size and state of the particles [9,10]. A progressive insidious bone resorption event associated with a well-functioning TJA is often referred to as periprosthetic osteolysis. The inability to diagnose the severity of bone defects at early stages eventually leads to bone destruction in the vicinity of implants, requiring early surgical interventions [11].

The pathogenesis of implant loosening is still not clear, but genetical, biological, and mechanical factors might be the contributing factors for implant-induced osteolysis [12,13]. Varied osteolytic responses to wear debris have highlighted the fact that genetic variation among the inflammatory and bone turnover signaling pathways are crucial factors for assessing the susceptibility of patients to osteolysis [3,14]. Anti-bone resorptive drugs such as bisphosphonates are available for pharmacological interventions for treating osteolysis. However, these drugs are useful only in the initial stages of osteolysis. Thus, in the absence of any regime of treatment, researchers are focusing on the cellular and molecular levels to study this multifactorial disease state and understand the molecular mechanism. A detailed insight into the molecular event could help in identifying any novel therapeutic targets.

The generation of wear particles from articular surfaces of a prosthesis forms a granulomatous periprosthetic membrane, which is abundant in macrophages, fibroblasts, chondrocytes, lymphocytes, endothelial cells, mesenchymal stem cells (MSCs), and implant-derived wear particles [15,16]. The majority of these cells can phagocyte the wear particles. Wear-debris-stimulated macrophages are the most prominent cells, which upon phagocytosing the submicron size wear particle secrete a variety of pro-inflammatory cytokines (IL-6, IL-11. IL-8, IL-1β, TNF-α, etc.) and bone-resorbing mediators (matrix metalloproteinases—MMP-1, MMP-13, RANKL), mediating bone degradation [17,18]. Dendritic cells are other immune cells that are able to releases various pro-inflammatory cytokines in response to wear particles [19].

In granulomatous soft tissue membranes around bone–prosthesis interfaces that are separate from the monocyte or macrophage cell linage, fibroblasts such as fibroblast-like synoviocytes (FLS) are the other cell types that are in close contact with wear debris. Recently, the role of FLS in the pathogenesis of aseptic loosening has been acknowledged. The dynamic integrity and the composition of the synovial fluid and extracellular matrix of the joints is maintained by FLS [20,21]. FLS can cause destruction of the extracellular matrix of bone by secreting bone-resorbing MMPs, stromelysin, and collagenase in response to wear debris [22]. Moreover, in response to wear debris, FLS releases pro-inflammatory cytokines like IL-6, IL-8, IL-1β, TNF-α, MCP-1, and RANKL [23]. These factors have a vital role in elevating bone-resorbing processes such as osteoclastogenesis. Ti and its alloys are the most promising biomaterials, which have been widely used in various kinds of arthroplasty prostheses and dental implants. Due to the formation of a stable thin oxide layer on its surface, Ti has superior biocompatibility and excellent corrosion resistance properties [4]. The process of passivation or repassivation spontaneously forms an oxide film on its surface. However, poor tribological properties and weak fretting fatigue resistance due to their low hardness make Ti and its alloys less favorable under strained mechanical conditions [24]. Because of these characteristics, a substantial number of Ti particles are often observed in tissues nearby the failed implants [25], and an ever-increasing accumulation of Ti particles can be expected over time. Recently, FLS has been shown to contribute either in an autocrine or paracrine manner to the complex milieu of periprosthetic space. However, the mechanism by which Ti particles affect FLS leading to secretion pro-inflammatory cytokines remains elusive. Henceforth, the purpose of this study was to investigate the effect of Ti particles on the human FLS cell line and to investigate the probable mechanism by which it might affect or participate in the process of bone loss during periprosthetic osteolysis.

## 2. Materials and Methods

### 2.1. Preparation of Ti Particles

Ti particles for this study were purchased from Johnson Matthey Company (Ward Hill, MA, USA). About 86% of Ti particles were of <10 μm in diameter and were confirmed by histologic analysis. Initially, Ti particles were sterilized by an overheated process of 6 h at 180 °C and kept submerged for 48 h in 70% ethanol. Next, Ti particles were suspended and preserved in sterile phosphate-buffered saline (PBS). For this study, Limulus assay (E-TOXATE; Sigma Aldrich, St Louis, MO, USA) confirmed that particles were endotoxin-free.

### 2.2. Cell Culture

The human synovial cell line, SW982, was obtained from the American Type Culture Collection (Rockville, MD, USA). SW982 cells were grown and maintained in sterile Dulbecco’s modified Eagle’s medium (DMEM) at 37 °C in a 5% CO_2_ incubator. To make complete growth medium, 10% fetal bovine serum (FBS: Gibco, Thermo Fisher, Grand Island, NY, USA), 2 mM L-glutamine, 100 U/mL penicillin, and 100 U/mL streptomycin (Invitrogen, Carlsbad, CA, USA) were added to DMEM.

### 2.3. MTT Assay

The 3-(4,5-dimethylthiazol-2-yl)-2,5-diphenyl-tetrazolium bromide (MTT; Sigma Aldrich, St Louis, MO, USA) assay was performed to evaluate the viability of cells after giving stimulation of Ti particles to SW982 cells, as per our lab-established protocol. Cells were cultured into 96-well plates and incubated overnight until reaching the required confluency level. The next day, MTT reagent at a concentration of 5 mg/mL per well was added and further incubated for 3 to 4 h at 37 °C in a 5% CO_2_ incubator after removal of culture media. Dark purple formazan, produced as a result of viable cells with an active metabolism, was dissolved by adding 200 µL/well of dimethyl sulfoxide (DMSO). Finally, the optical density of each sample was read at 570 nm by using a plate-reading spectrophotometer.

### 2.4. Lactate Dehydrogenase Activity (LDH) Assay

Cytotoxicity detection kit (Roche Diagnostics, Indianapolis, IN, USA) was used to measure LDH released into the cell culture media from damaged cells. To perform LDH activity assays, 10 μL cell culture media was used and added into a new 96-well plate with 40 μL sterile PBS. Then, 50 μL of LDH reagent provided in the kit was added to each well. The plate was then kept in the dark for incubation (45 min). Stop solution was added into each well to stop the enzymatic reaction. Spectrophotometer at 490 nm wavelength was used to measure optical density. For positive control, complete cell lysate was used.

### 2.5. Protein Isolation and Western Blotting

RIPA (Radioimmunoprecipitation assay buffer) cocktail buffer supplement with protease inhibitors (Roche Diagnostics, Indianapolis, IN, USA) was added into the wells for cell lysis. The protein concentration of each sample was evaluated using a protein assay kit (Bio-Rad Laboratories, Hercules, CA, USA). As per our lab manual, SDS-polyacrylamide gel electrophoresis was performed with the isolated total protein [26]. The membrane was probed with the required primary antibodies against Cox-2, IκBα, phospho-extracellular signal-regulated kinase (pERK), ERK, phospho-c-Jun N-terminal kinase (pJNK), JNK, phospho-p38 (p-p38), and p38 (Cell Signaling Technology, Danvers, MA, USA). As a loading control, β-actin antibody (Santa Cruz Biotechnology, Dallas, TX, USA) was used. Blots were rinsed twice with 10 mM Tris-HCl, 50 mM NaCl, 0.25% Tween 20 (TBST) prior to secondary antibody treatment. Target proteins on blots were detected with horseradish-peroxidase-conjugated secondary antibody and visualized using treating chemiluminescence reagents (Bionote, Inc., Gyeonggi-do, Korea).

### 2.6. RNA Isolation and Real-Time RT-PCR

Total RNA was harvested from the cells by adding Trizol reagent (Invitrogen). The quality and integrity of RNA samples were evaluated carefully before performing RT-PCR. For synthesized first-strand cDNA, total RNA (2 µg) was used with SuperScript II (Invitrogen, Carlsbad, CA, USA). Each PCR blend contained one-tenth of the cDNA and EXPRESS SYBR green qPCR Supermix (BioPrince, Seoul, Korea). In the real-time PCR analysis, the thermal cycle reaction included about 10 min preheating of samples at 95 °C and amplification of 50 cycles at 95 °C for 20 s, 60 °C for 20 s, and 72 °C for 25 s. The relative mRNA expressions of each selected target gene were standardized and normalized to the housekeeping gene, glyceraldehyde 3-phosphate dehydrogenase (GAPDH). For quantification, the ΔΔCT method was used. The sequences of primers used for RT-PCR are listed in Table 1. 

### 2.7. Luciferase Assay

SW982 cells were cultured in a 24-well plate at a confluence of 4 × 10^5^ cells. Then, cells were transfected with 100 ng AP-1 plasmid construct (Addgene, Cambridge, MA, USA) and another Renilla luciferase thymidine construct (Invitrogen) by utilizing Genefectine transfection reagent (Genetrone Biotech Co., Seoul, Korea), as per the manufacturer’s recommendation. Ti particles were treated for 24 h to transfect the cells. The luciferase activity was assessed with cell lysate of SW982 cells using a dual-luciferase assay kit (Promega, Sunnyvale, CA, USA). Luminometer (Glomax, Promega) was utilized to measure luciferase activity. Every sample reading was standardized and normalized with Renilla luciferase activity.

### 2.8. ELISA

ELISA kit was used for the quantitative measurement of pro-inflammatory cytokines, such as IL-6 (Ab Frontier, Seoul, Korea), IL-1β (Ab Frontier), and TNFα (ABclonal Biotechnology, Woburn, MA, USA), in the cell culture medium. ELISA was performed as per the manufacturer’s protocol.

### 2.9. Statistical Analysis

All the statistical data associated with this study were analyzed by Graphpad Prism 8.2 (San Diego, CA, USA) and assessed by a two-tailed Student’s *t*-test. Values measuring *p* < 0.05 were considered to indicate statistical significance.

## 3. Results

### 3.1. Ti Particles Induce Inflammation in FLS

**Figure 1:**
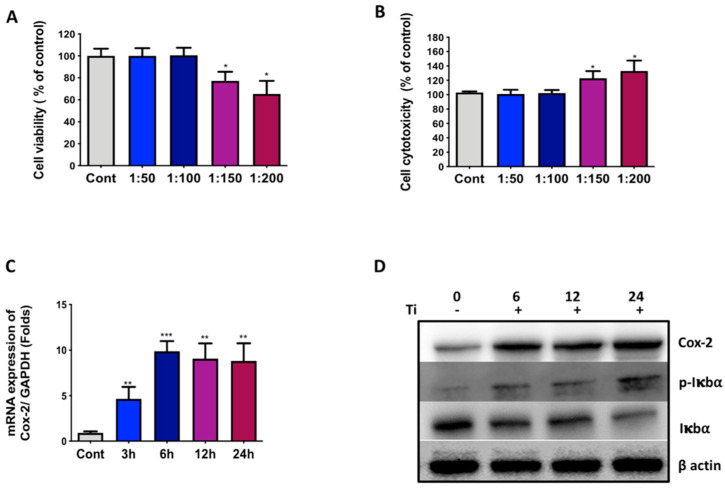
Ti particle effects on SW982 cell line. Ti particles were treated with SW982 cells for 24 h in a dose-dependent manner (cell-to-particle ratios of 1:50, 1:100, 1:150, and 1:200). The control (Cont.) group was treated with PBS only. Cell viability and cell cytotoxicity were analyzed by (**A**) MTT and (**B**) LDH activity assay, respectively. (**C**) RT-PCR analysis after the treatment with Ti particles (cell-to-particle ratio of 1:100) of SW982 at different time points (0 to 24 h) displayed an increase in the mRNA expression level of Cox-2. (**D**) Quantification of protein by Western blotting after 0, 6, 12, and 24 h of Ti particle treatment (cell-to-particle ratio of 1:100) of SW982 cells showed increased expression levels of Cox-2 and p-Iκbα. At the same time, an instability of Iκbα was observed. The results are demonstrated as means ± standard deviations (SDs) of three different independent experiments. Note: * *p* < 0.05, ** *p* < 0.01, and *** *p* < 0.001 indicate significant differences from the control group.

Initially, any effect of Ti particles as wear debris on fibroblast-like synoviocytes (FLS) was analyzed. In this study, the SW982 synovial cell line was used to depict FLS-like characteristics [27]. Several cell-to-Ti particles ratios were used for the treatment of SW982 for 24 h, and any effects of Ti particles on the cell viability and cell toxicity were analyzed by MTT and LDH assays, respectively. The results demonstrated that until reaching a ratio of 1:100 (cells to Ti particles), there was no effect on the cell viability or cell cytotoxicity of SW982 cells (Figure 1A,B). Hence, a ratio of 1:100 was used for further experiments. As evidenced by RT-PCR results, a time-dependent treatment with Ti particles of SW982 cells showed induction in the mRNA expression pro-inflammatory marker, Cox-2 (Figure 1C). After 3 h of treatment, a 5-fold increase in Cox-2 mRNA was observed, while after 6 h and until the observed time point a 24 h, nearly 10-fold mRNA induction was recorded (Figure 1C).

Further, Ti particles were treated with SW982 cells for 6, 12, and 24 h, and the expression level of Cox-2 expression was analyzed by Western blotting. The results showed that after 6 h of treatment, an induction in the expression level of Cox-2 was observed until 24 h compared to the control (Figure 1D). Moreover, phosphorylation of IκBα was observed after 6 h of Ti particle treatment until 24 h compared to the control, implicating activation of NFκB signaling activity. The instability of IκBα further verified the activation of NFκB signaling after the treatment of Ti particles to SW982 cells. Taken together, the results here clearly demonstrate the induction of Cox-2 and NFκB signaling pathways in FLS after Ti particle treatment for FLS.

### 3.2. Ti Particles Induced the Expression of Pro-Inflammatory Cytokines in FLS

**Figure 2:**
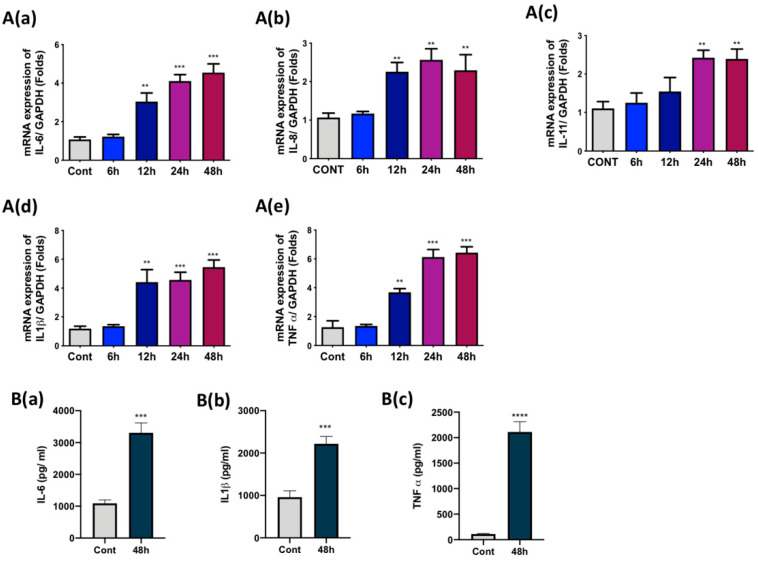
Effects of Ti particles on the secretion of pro-inflammatory cytokines from SW982 cells. Ti particles were used to treat (cell-to-particle ratio of 1:100) SW982 at several time points until 48 h. (**A**) RT-PCR analysis showed increased mRNA expression of (**a**) IL-6, (**b**) IL-8, (**c**) IL-11, (**d**) IL-1β, and (**e**) TNF α. The mRNA expression of each targeted gene was normalized to glyceraldehyde 3-phosphate dehydrogenase (GAPDH). (**B**) ELISA results demonstrated enhanced secretion of pro-inflammatory cytokines such as (**a**) IL-6, (**b**) IL-1β, and (**c**) TNF α in Ti-particle-stimulated SW982 medium. The results are demonstrated as means ± SDs of three independent experiments. In the graphical representations, ** *p* < 0.01, *** *p* < 0.001, and **** *p* < 0.0001 indicate significant differences from the control group.

As we observed an induction of the Cox-2 and NFκB signaling pathway, an analysis of the release of cytokines in FLS after the treatment with Ti particles was required. For this, SW982 cells were treated with Ti particles for 6, 12, 24, and 48 h, and mRNA was collected. RT-PCR analysis demonstrated an increase in the mRNA expression of IL-6, IL1β, TNFα, IL-8, and IL-11 in SW982 cells after 12 h of treatment (Figure 2A–E). However, the mRNA expression levels at 12 h varied for each cytokine—IL-6 showed 3-fold, IL1β showed 4.5-fold, TNFα showed 3.5-fold, IL-8 showed 2-fold, and IL-11 showed 1.5-fold expression. Maximum mRNA expression was observed at 48 h for most of the cytokines—IL-6 showed 4.5-fold, IL1β showed 5.5-fold, TNFα showed 6-fold, IL-8 showed 2-fold, and IL-11 showed 2.5-fold expression. Since maximum induction of more than 4-fold or above was observed for IL-6 (4.5 fold), IL1β (5.5 fold), and TNFα (6.5 fold), we tried to analyze the secreted amounts of these cytokines in the culture medium. ELISA results showed that after 48 h of Ti particle treatment of SW982 cells, elevated amounts of IL-6 (3300pg/mL), IL1β (2218 pg/mL), and TNFα (2111 pg/mL) were observed in the culture medium (Figure 2B(a–c)).

### 3.3. Ti Particles Activate ERK and JNK Signaling Pathways in FLS

**Figure 3:**
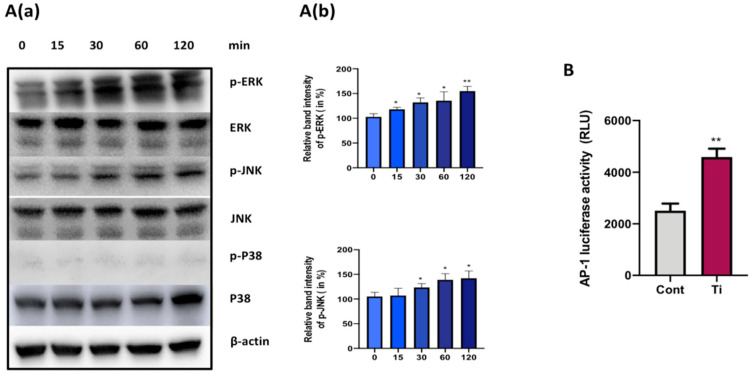
Effect of Ti particle on the activation of MAPKs in SW982 cells. (**A**(**a**)) Representative Western blots showing protein levels of total and phosphorylated forms of extracellular signal-regulated kinase (ERK) and c-Jun N-terminal kinase (JNK) and P38 mitogen-activated protein kinases (MAPKs) after treatment with Ti particles (cell-to-particle ratio of 1:100) of SW982 cells for 15, 30, 60, and 120 min. Here, β-actin was taken as control. (**A**(**b**)) Fusion FX software was utilized for quantitative densitometric analysis of the proteins. (**B**) AP1-luc reporter plasmids were transfected to SW982 cells for 24 h and luciferase activity was analyzed. Renilla luciferase activity was used for normalization. The results are demonstrated as means ± SDs of three independent experiments. In the graphical representations, * *p* < 0.05 and ** *p* < 0.01 indicate significant differences from the control group.

Any activation of mitogen-activated protein kinase (MAPKs) in FLS via treatment with Ti particles was assessed at early time points by Western blotting. Ti particle stimulation of SW982 cells induced the phosphorylation of ERK MAPKs at an early time point of 15 min until 2 h of treatment (Figure 3). Quantification of Western bands showed a significant increment in the phosphorylation of ERK MAPKs (Figure 3A(a)). Similarly, phosphorylation of JNK was also observed after 15 min of Ti particle treatment until 2 h. In the case of JNK MAPKs, a significant increase in the phosphorylation of JNKs was recorded after 30 min of Ti particle stimulation of SW982 cells (Figure 3A(b)). However, any phosphorylation of p38 was not observed after the Ti particle treatment for the above time course. Moreover, the increased luciferase reporter activity of the activator protein-1 (AP-1) construct confirmed the activation of MAPK signaling pathways in Ti-particle-treated SW982 cells. This implies that the ERK and JNK signaling pathway might be involved in the secretion of inflammatory cytokines in Ti-particle-treated SW982 cells.

### 3.4. Co-Inhibition of ERK and JNK Signaling Pathways Suppressed Secretion of IL-6, IL1β, and TNFα from FLS

**Figure 4:**
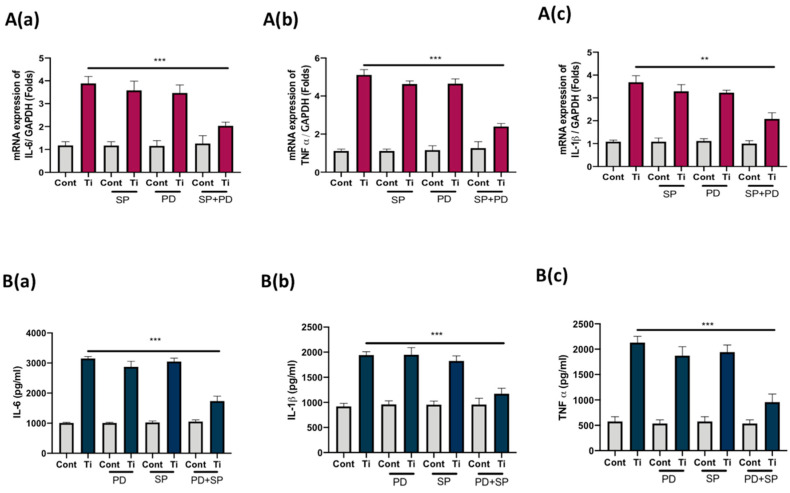
Co-inhibition of ERK and JNK MAPKs suppresses the expression of pro-inflammatory cytokines in SW982 cell line. Ti particles were used to treat (Cell-to-particle ratio of 1:100) SW982 cells prior to incubation (30 min) with either PD98059 (5 µM), SP600125 (5 µM), or PD98059 along with SP600125 for 24 h. RT-PCR analysis showing the mRNA expression of (**A(a)**) IL-6, (**A(b)**) IL-1β, and (**A(c)**) TNF α after co-inhibition of ERK and JNK MAPKs. The mRNA expression of each targeted gene was normalized to GAPDH. (**B**) ELISA results demonstrated secretion levels of (**B(a)**) IL-6, (**B(b)**) IL-1β, and (**B(c)**) TNF α after co-inhibition of ERK and JNK MAPKs. The results are presented as means ± SDs of three independent experiments. In the graphical representations, ** *p* < 0.01 and *** *p* < 0.001 indicate significant differences from the control group.

To define the role of activated ERK and JNK pathways in the secretion of inflammatory cytokines in Ti-particle-stimulated FLS, we tried to inhibit the respective MAPKs with their specific inhibitors and analyzed the secretion of cytokines from FLS. SW982 cells pre-incubated with either PD98059 (5 µM), SP600125 (5 µM), or PD98059 along with SP 600,125 for 30 min were treated with Ti particles for 48 h. The concentration of PD98059 + SP 600,125 utilized for inhibition was enough to inhibit the activity of ERK and JNK in SW982 cells. The mRNA was harvested after 48 h of treatment and screened for the expression of IL-6, IL1β, and TNFα. Treatment alone with either PD98059 or SP600125 was not sufficient to block the secretion of IL-6, IL1β, and TNFα from Ti-particle-stimulated SW982 cells. However, co-inhibition of ERK and JNK MAPKs resulted in suppression of mRNA expression of IL-6, IL1β, and TNFα in Ti-particle-stimulated SW982 cells (Figure 4A(a–c)). Moreover, ELISA results also demonstrated that the co-inhibition of ERK and JNK MAPKs in Ti-particle-stimulated SW982 cells was able to significantly reduce the secretion of IL-6, IL1β, and TNFα (Figure 4B(a–c)).

## 4. Discussion

The prostheses used in TJA have to mimic natural functional roles, and thus have to withstand mechanical and chemical challenges and provide a durable, smooth sliding surface for painless stable movement over a period of time [28]. All total joint replacement implants are exposed to particulates from the bearing surfaces, fabrication wear debris, and additional by-products of the different materials cast in the surgical reconstruction [29]. Aseptic loosening of implants usually follows after periprosthetic osteolysis in the majority of cases, which unfortunately is asymptomatic in nature for a long time. It may be observed that a fibrous membrane, irregularly organized and resembling synovial tissue, can form around the bone–prosthesis interface post-operatively [30]. This synovial-like interfacial membrane is primarily composed of macrophages and fibroblasts and enables the expansion of particle disease across the prosthetic tissue via the joint fluid. Numerous researchers have admitted that the FLS might play a decisive role during the initial stage of the biological reaction of the body to wear debris generation [7,31,32]. Hence, this study aimed to assess the response of FLS to Ti particles as wear debris and investigate the probable mechanism behind this. Usually, primary FLS from the synovial membrane is the preferred choice to study the physiological response of FLS to inflammatory signals. However, primary FLS are associated with certain drawbacks, such as difficulties in harvesting and establishing the culture, due to heterogenous clones, a lack of reproducibility, and varying outcomes due to non-standardized patient tissue samples. Thus, herein SW982 cells were utilized in the human synovial cell line. SW982 cells have been shown to possess characteristic features similar to FLS in synovium and have been successfully used to study rheumatoid arthritis [27]. Elevated Cox-2 expression along with the activation of NFκB signaling pathway in SW982 confirmed an inflammatory response to Ti particle treatment (Figure 1C,D). A cell-to-Ti particle ratio of 1:100 was sufficient to induce this inflammatory response without affecting the cell viability or toxicity of SW982 cells (Figure 1A,B).

In response to wear debris, FLS has been shown to secrete various pro-inflammatory cytokines in the synovium [33,34]. Since Ti particles induced Cox-2 expression and activated the NFκB signaling pathway in SW982, we expected an induction of the release of pro-inflammatory cytokines from Ti-particle-stimulated SW982 cells. Our results showed increased mRNA expression of IL-6, IL-8, IL-11, IL-1β, and TNFα in 48 h Ti-particle-treated SW982 cells (Figure 2A(a–e)). However, among these, the induction of cytokines IL-6, IL-1β, and TNFα was more than 5-fold. Hence, we analyzed the amounts of these cytokines in the medium using ELISA. The results showed elevated amounts of IL-6 (~3500 pg/mL), IL-1β (~2400 pg/mL), and TNFα (~2300 pg/mL) in the medium containing 48 h Ti-particle-treated SW982 cells (Figure 2B(a–c)). Induction of cytokines at mRNA and protein levels confirmed our inflammatory FLS model of SW982 cells, as well established the release of inflammatory mediators in the medium containing Ti-particle-stimulated SW982 cells. As discussed, FLS are crucial for the maintenance of the synovium, as they contribute to the extracellular matrix state of the synovial membrane. Joints are encapsulated with synovium, which is responsible for providing structural support, lubrication abilities, and nutrition to the cartilage. Hence, it has been clinically suggested that during total hip replacements, some of the synovium could be retrieved to lessen the friction amongst the different parts of the implants [35]. However, considering the release of pro-inflammatory cytokines by FLS in response to wear debris, a retrieval of synovium for lubrication purposes should be reconsidered by the clinicians after replacement surgeries.

Activation of MAPKs by wear particles is associated with induced inflammatory responses in various cell types [36,37,38]. Wear debris has been shown to stimulate intracellular signaling pathways such as MAPKs, leading to the secretion of pro-inflammatory cytokines. For example, Ti particles have been shown to activate MAPKs in macrophages to induce the secretion of various cytokines [39]. Hence, we analyzed any activation of MAPKs in Ti-particle-treated FLS at early time points (Figure 3). The results demonstrated the activation of ERK and JNK signaling pathways after 15 min of stimulation of SW982 cells with Ti particles. Activation of the ERK signaling pathway is associated with the release of IL-6 in osteoprogenitors [40], while JNK MAPKs have been shown to mediate the release of pro-inflammatory cytokines in macrophages [41]. Concurrently, we found that Ti particles have the ability to activate ERK and JNK signaling pathways in the case of FLS.

The P38 MAPK signaling pathway has been shown to be activated by Ti particles in mouse osteoclasts, and its inhibition is shown to be associated with the downregulation of inflammatory osteolysis [42]. However, no activation of p38 MAPK was not observed in SW982 cells (Figure 3). Likewise, no activation of p38 by Ti particles has been reported in osteoblasts [36]. It appears that p38 signaling activation depends on the cell type, which might be essential for osteoclasts and macrophages, contributing to the inflammatory osteolysis.

In order to investigate any relationship of the activation of ERK and JNK signaling pathways with the release of pro-inflammatory cytokines (IL-6, IL-1β, and TNFα) in SW982 cells, we tried to inhibit the ERK and JNK MAPKs with their specific inhibitors (PD98059 for ERK and SP600125 for JNK) and analyzed any effects on the induction of mRNAs and secretion of IL-6, IL-1β, and TNFα from SW982 cells. Inhibition of either ERK or JNK MAPKs did not affect the release of IL-6, IL-1β, and TNFα in SW982 cells. However, a co-inhibition of both MAPKs (ERK and JNK MAPKs) significantly reduced the cytokines’ mRNA induction, as well as the secretion in the cellular medium of SW982 cells (Figure 4A,B). Thus, it can be assumed that in the case of FLS, Ti particles activate ERK and JNK MAPKs to induce secretion of pro-inflammatory cytokines, such as IL-6, IL-1β, and TNFα. Enhanced secretion of these cytokines might further aggravate the bone resorbing process at the site of wear-debris-induced inflammatory osteolysis. For instance, TNF-α from macrophages has been shown to affect the osteogenic ability of osteoprogenitors, IL-6 acts as a chemokine to attract macrophages at the site of inflammation, and IL-1β is known to induce osteoclasts formation [40,43]. Thus, inhibition of the ERK and JNK MAPKs might be able to reduce the secretion of IL-6, IL-1β, and TNFα in FLS, which in turn could help in suppressing the hyper immunological cellular damage around the implants.

However, as the inhibition of ERK and JNK MAPKs could not completely block the secretion of cytokines from Ti-particle-stimulated FLS, this raises the possibility of involvement of other signaling pathways. For instance, the JAK-STAT signaling pathway has been reported to be involved with the secretion of pro-inflammatory cytokines during wear-debris-induced osteolysis [44]. Moreover, we have observed the induced expression of Cox-2 and activation of the NFκB signaling pathway after the treatment of SW982 cells with Ti particles. In synovial fibroblasts, crosstalk between JNK, ERK, and Cox-2 has been suggested under inflammatory conditions [45]. It appears that Ti particles stimulate ERK and JNK MAPKs in FLS, and might have an interaction with the NFκB signaling pathway downstream for the release of pro-inflammatory cytokines. However, further studies would be required to delineate the crosstalk among these signaling pathways, which appears otherwise complicated in inflammatory conditions.

## 5. Conclusions

The study provides evidence of the fact that the Ti particles can stimulate FLS to secrete a significant amount of pro-inflammatory cytokines such as IL-6, IL-1β, and TNFα in the synovium. For this, Ti particles activate ERK and JNK MAPKs at every early time point to initiate the release of these cytokines. Co-inhibition of ERK and JNK MAPKs could prevent the excessive release of these bone-resorbing cytokines from FLS. Thus, targeting ERK and JNK MAPKs might provide a therapeutic option for containing the release of the pro-inflammatory cytokines from FLS, and could help to protect the elevated bone resorption, as observed during inflammatory conditions such as rheumatoid arthritis and periprosthetic osteolysis.

## Figures and Tables

**Table 1:** Primers for real-time RT-PCR.

Target	Forward Primer (5′–>3′)	Reverse primer (3′–>5′)
Cox-2	CCAAATCCTTGCTGTTCCCACCCAT	GTGCACTGTGTTTGGAGTGGGTTT
IL-6	CCAGCTATGAACTCCTTCTC	GCTTGTTCCTCACATCTCTC
IL-8	AAGAAACCACCGGAAGGAACCATCT	AGAGCTGCAGAAATCAGGAAGGCT
IL-11	AGATATCCTGACATTGGCCAGGCA	ACTTCAGTGATCCACTCGCTTCGT
IL-1β	AACCAGGCTGCTCTGGGATTCTCTT	ATTTCACTGGCGAGCTCAGGTACT
TNF-α	AAGGACGAACATCCAACCTTCCCAA	TTTGAGCCAGAAGAGGTTGAGGGT
GAPDH	TCGACAGTCAGCCGCATCTTCTTT	ACCAAATCCGTTGACTCCGACCTT
